# An Insight Into the Modulatory Effects and Mechanisms of Action of *Phyllanthus* Species and Their Bioactive Metabolites on the Immune System

**DOI:** 10.3389/fphar.2019.00878

**Published:** 2019-08-07

**Authors:** Ibrahim Jantan, Md. Areeful Haque, Menaga Ilangkovan, Laiba Arshad

**Affiliations:** ^1^School of Pharmacy, Faculty of Health and Medical Sciences, Taylor’s University, Lakeside Campus, Subang Jaya, Malaysia; ^2^Department of Pharmacy, International Islamic University Chittagong, Chittagong, Bangladesh; ^3^Fairview International School, Kuala Lumpur, Malaysia; ^4^Department of Pharmacy, Forman Christian College (A Chartered University), Lahore, Pakistan

**Keywords:** *Phyllanthus* species, ethnomedicine, phytochemicals, immunosuppressive effects, immunomodulators

## Abstract

*Phyllanthus* species (family; *Euphorbiaceae*) have been intensively studied for their immunomodulating effects due to their wide-ranging uses to treat immune-related diseases in indigenous medicine, which are primarily lack of scientific basis. The focuses of this review are on the significance of *Phyllanthus* species and their bioactive metabolites particularly corilagin (**1**), geraniin (**2**), gallic acid (**3**), phyllanthin (**4**), hypophyllanthin (**5**), ellagic acid (**6**), phyltetralin (**7**), niranthin (**8**), catechin (**9**), quercetin (**10**), astragalin (**11**), and chebulagic acid (**12**) in the modulation of both innate and adaptive immune systems through various mechanisms and their possible therapeutic benefits for treatment of immune-related diseases. We have compiled all significant findings published in the literature, and the data were analyzed critically to provide perspectives and directions for future research for the plants as a prospective source of novel immunomodulating agents. Various *Phyllanthus* species particularly *Phyllanthus amarus*, *Phyllanthus emblica*, *Phyllanthus niruri*, and *Phyllanthus urinaria* have been documented to possess significant immunomodulatory effects. However, the possible challenges encountered by the application of extracts of various *Phyllanthus* species and their bioactive constituents as immunomodulators need to be addressed. Most reports on the biological and pharmacological studies of the plants were based on crude extracts. The extracts were not chemically characterized, and the contributions of their chemical constituents to the bioactivities were not identified. The underlying mechanisms involved in the immunomodulatory effects of the *Phyllanthus* species were not indepthly studied due to limitations in terms of design, conduct, and interpretation. Extensive experimental and preclinical studies on the immunomodulating potential of *Phyllanthus* species should be carried out to provide sufficient data to prove that their traditional uses are inherently effective and safe and will allow clinical trials to be pursued for their further development as therapeutic agents to treat immune-related disorders.

## Introduction

**Figure 1:**
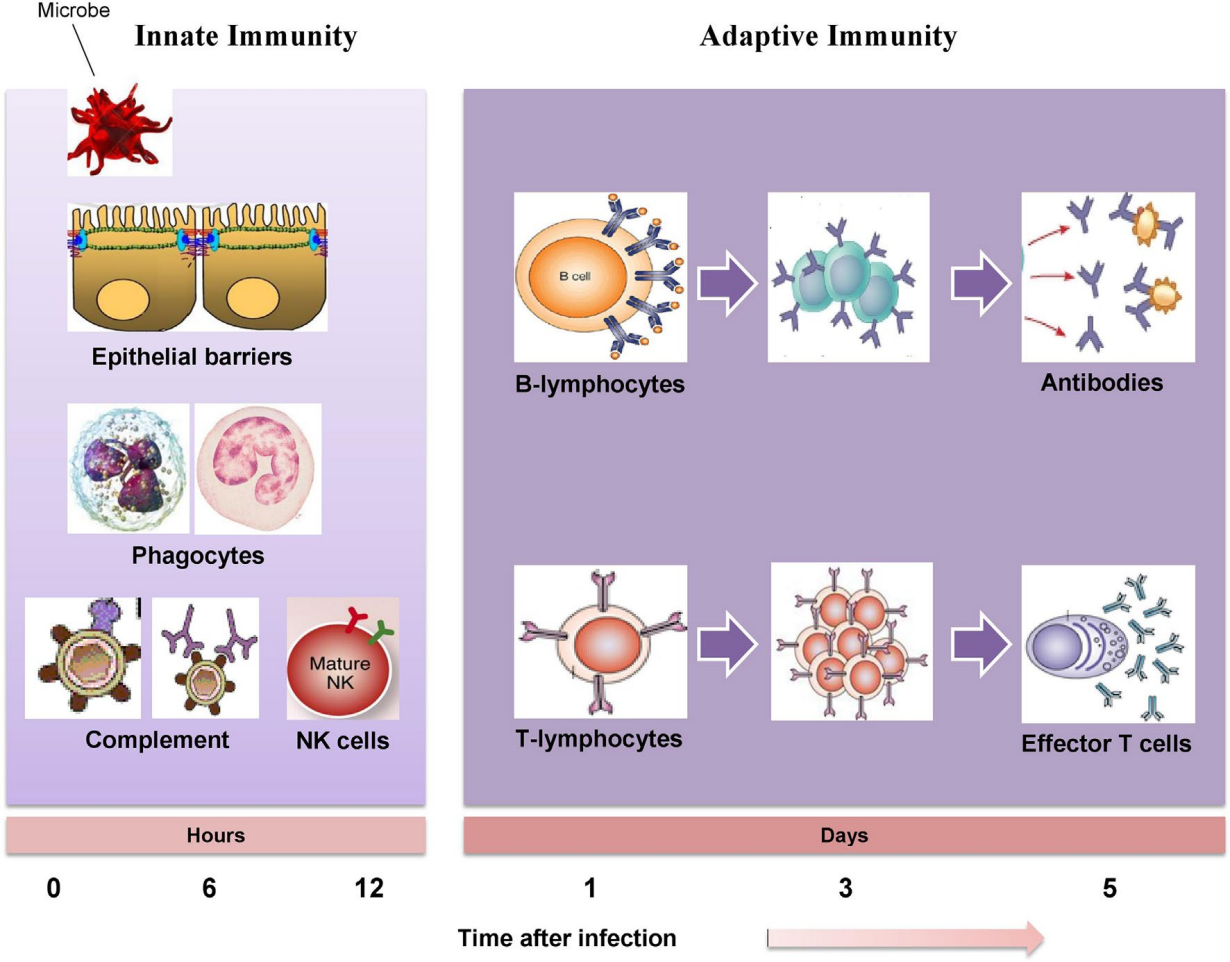
The mechanisms of innate immunity providing initial defense against invading pathogen followed by adaptive immune responses develop later and consist of activation of lymphocytes.

The immune system is composed of white blood cells (neutrophils, lymphocytes, macrophages, and monocytes) and specialized immune molecules (cytokines, antibodies, and complement proteins) that have developed to bring about resistance to infections. Synchronized interaction of several immune cells with these molecules induces an apt immune response (Chaplin, 2010). The immune system governs numerous interconnecting signaling events of microbial detection, microbial clearance, inflammation, cell and tissue damage and death, as well as wound healing. The immune system acts as a defense system that protects the host from invading pathogen and requires the timely interaction of several immune cells and crosstalk within the specific tissue microenvironment to sustain homeostasis (Mak and Saunders, 2005). An adequate redox balance of immune cells has to be maintained; a failure of which may lead to the pathogenesis of immune-related diseases including infections, cancers, inflammatory bowel diseases, dermatitis, metabolic syndrome, and asthma. There are three core elements essential in providing an effective immune response which include the recognition of a diverse array of pathogens and phagocytosis of pathogens as soon as they are recognized and sparing the tissue of host (Stephens et al., 2002). The immune responses of the human body are categorized into the innate immunity and the adaptive immunity. The innate immunity is a non-specific immune system which provides the initial protection against infections (Greenberg and Grinstein, 2002) while the adaptive immunity is a specific or acquired immune system which develops gradually and specifically adapted for the invading pathogens. The presence of memory cells enhances the response to repeated exposure to alike antigen (Janeway et al., 2005). **Figure 1** clearly illustrates the host defense mechanisms, consisting of the innate immune response (providing early protection against invading pathogens), and the adaptive immune response (mediating later but more effective defense against infections).

Studies have shown that most immune-associated diseases, such as pathogen-mediated infectious diseases, allergic diseases, and cancers are associated with inflammation. The process of inflammation, induced by harmful stimuli or conditions could be a critical immune response. Inflammation is an underlying mechanism to a wide variety of physiological and pathological processes that facilitate repairing of damaged tissues and maintains the body homoeostasis (Ademokun and Dunn‐Walters, 2001). In principle, the efficiency of immune system relies on the dynamic interplay between antigen and a network of immunologically competent cells as there are several mechanisms by which the usual immune restoration process can go wrong. An effective immune restoration process may be desirable in two different situations: 1) insufficient immune function and 2) overly expressed immune function. There are multiple pathways utilized by the immune system in restoring immune balance (Andhavarapu and Roy, 2013). Immunomodulators have been used to restore immune imbalance. Immunomodulators are natural, biological, or synthetic substances that can increase, inhibit, or modulate both innate and adaptive functions of the immune system. The interest in immunomodulators is increasing due to a greater awareness that many human diseases are associated with immune abnormalities of pathological significance and an expanding knowledge of immune function (Ben-Efraim, 2001). Clinically, immunomodulators are categorized into three diverse classes including immunosuppressant, immunostimulant, and immunoadjuvant. The increasing interest in immunomodulators leads to the discovery and development of diverse array of recombinant, synthetic, and natural immunomodulators for the improvement of disease resistance and to handle immune disorders of the host by optimum modulation of immune system (Patil et al., 2012).

Although many synthetic immunomodulatory drugs with various mechanisms of action have been discovered and developed, they failed to be successful clinically due to their toxicity, less bioavailability, and stability problem. Medicinal herbs and their active metabolites deliver alternative potential to ongoing therapy for a wide array of immunological disorders by modulation of the immune response (Mohamed et al., 2017). Many medicinal herbs have long been utilized in different cultures and civilizations such as Chinese, Ayurvedic (India), Kampo (Japan), and UnaniTibb (South Asia) as traditional therapies for a spectrum of immune-related disorders without any scientific and clinical evidence supporting their uses (Haranaka et al., 1985; Singh et al., 2002). Investigations involving the immune system are currently one of the fast developing research areas as immune system acts as the pathological basis of multiple diseases. In this context, a spectrum of studies has been carried out to evaluate and select medicinal plants and natural compounds for drug discovery programs. Research to discover natural products as drug candidates for development of immunomodulatory agents has gained momentum as they offer safer alternatives to conventional therapies (Mohamed et al., 2017). Bioactive metabolites isolated from various medicinal plants can be categorized into several subclasses based on their chemical structures: carbohydrates, terpenes, steroids, phenolics of different biogenetic classes, amino acids, proteins, alkaloids, glycoproteins, peptides, and coumarins. Identification of the isolated compounds is also crucial in the evaluation of their potential effect on the regulation of immune system through various pathways. Among the natural immunomodulators in clinical studies are andrographolide, apigenin, asiaticoside, baicalein, berberine, boswellic acid, curcumin, and resveratrol (Ammon, 2006; Burgos et al., 2009; Jurenka, 2009; Sarris et al., 2013; Tome-Carneiro et al., 2013; Jantan et al., 2015; Kumar et al., 2015).

This review focuses on the significance of *Phyllanthus* species and their bioactive metabolites in the regulation of both innate and adaptive functions of the immune system through various mechanisms and their possible therapeutic benefits for treatment of immune-related diseases. There are many reported studies on the chemical and pharmacological properties of some *Phyllanthus* species, as well as a few clinical studies. The main focus of this review is on the immunomodulatory effects of several *Phyllanthus* species, i.e., *Phyllanthus amarus*, *Phyllanthus urinaria*, *Phyllanthus emblica*, *Phyllanthus niruri*, *Phyllanthus acidus*, *Phyllanthus fraternus*, *Phyllanthus reticulatus*, and *Phyllanthus simplex* that are extensively used in indigenous medicine for healing immune-related diseases and their reported immunomodulating properties in cell, animal, or human studies. Geographical distribution, ethnomedicinal values, and phytochemistry of the *Phyllanthus* species are also reviewed briefly. Extensive literature searches have been performed to gather data from the Science Direct, Institute for Scientific Information (ISI)-Web of Science, PubMed, Google Scholar, Scopus, scientific reports in Frontiers, Wiley Online Library, Elsevier, Springer, ACS Publications Today, Taylor & Francis, and others, and books published in the past few decades. The gathered data were analyzed critically to provide the strategies and perspectives for further research on *Phyllanthus* species as a potential source of new immunomodulating agents.

## Taxonomy and Distribution

**Figure 2:**
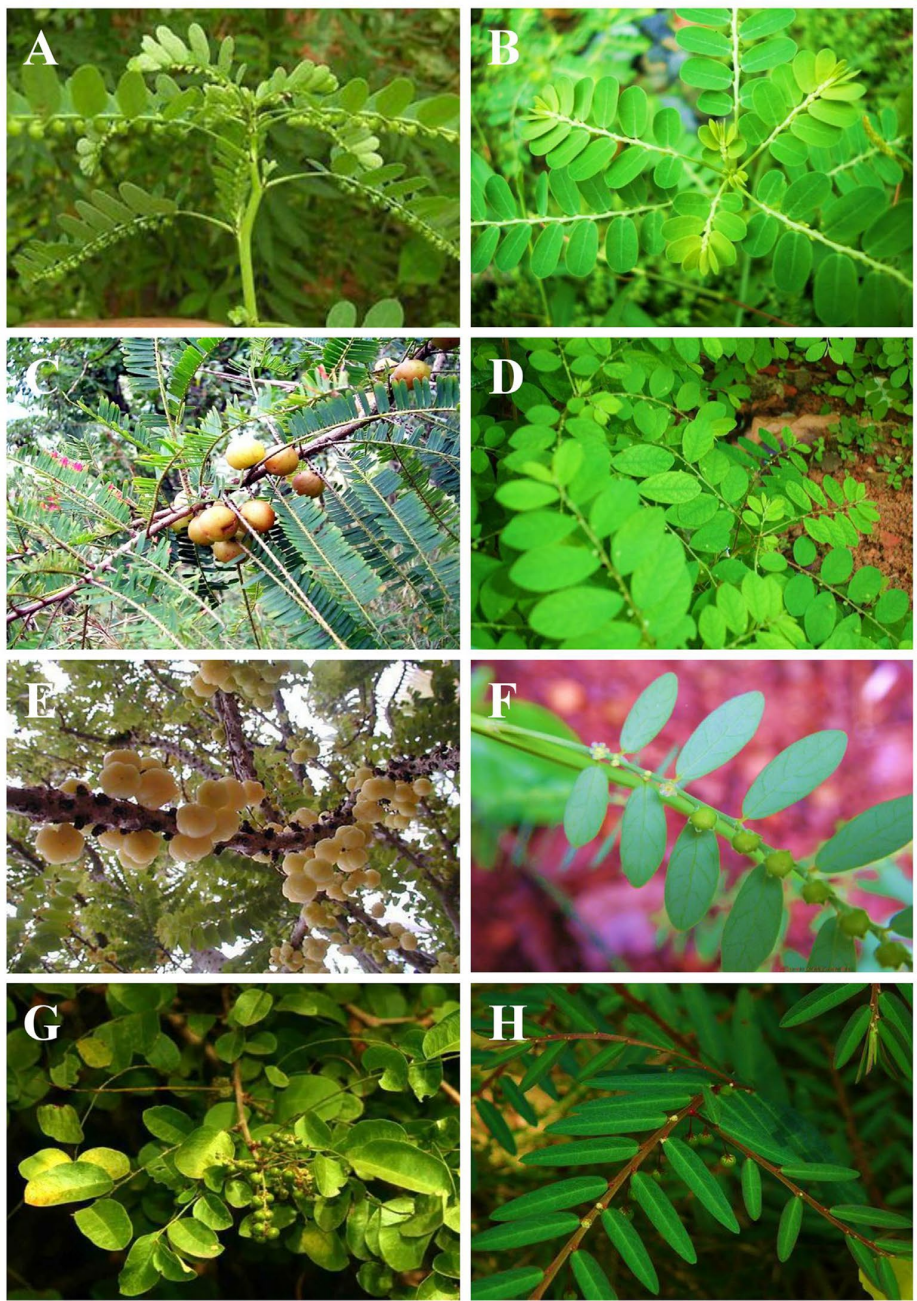
*Phyllanthus* species (Phyllanthaceae); **(A)** *P. amarus*, **(B)** *P. urinaria*, **(C)** *P. emblica*, **(D)** *P. niruri*, **(E)** *P. acidus*, **(F)** *P. fraternus*, **(G)** *P. reticulatus*, and **(H)** *P. simplex*.

*Phyllanthus* is the largest genus of Phyllanthaceae family. It is further subdivided into 11 subgenera including Gompidium, Bortryanthus, Conani, Cicea, Emblica, Ericocus, Isocladus, Kirganelia, Phyllanthodendron, Xyllophylla, and Phyllanthus. There are 1,270 species of *Phyllanthus* distributed throughout most of the subtropical and tropical regions including tropical Africa, tropical America, Oceania, and Asia (Hoffmann et al., 2006). About 200 *Phyllanthus* species are widely distributed in tropical America, mainly in the Caribbean and Brazil (Unander et al., 1995). There are 20 species of *Phyllanthus* commonly found throughout Malaysia which include *P. amarus*, *P. albidiscus*, *P. chamaepeuce*, *P. columnaris*, *P. debilis*, *P. elegans*, *P. emblica*, *P. filicifolius*, *P. gomphocarpus*, *P. gracilipes*, *P. oxyphyllus*, *P. pachyphyllus*, *P. pulcher*, *P. reticulates*, *P. ridleyanus*, *P. roseus*, *P. sikkimensis*, *P. urinaria*, *P. virgatus*, *and P. watsonii* (Burkill, 1966). Of all these, *Phyllanthus* species, *P. amarus*, *P. emblica*, *P. urinaria*, *P. niruri*, *Phyllanthus acidus L.*, *P. fraternus*, *P. reticulatus*, and *P. simplex* (**Figure 2**), are discussed in this review due to their wide use in traditional medicine to heal immune-related diseases and their reported immunomodulating properties in cell, animal, or human studies.

*P. amarus* Schum and Thonn (synonym: *P. niruri* Auct). is found in tropical and subtropical regions. It is an erect annual herb, usually found in fields, grasslands, and forests and is erect annual herbs, 10–60 cm tall. Leaves distichous, flowers small in leaf axils, 1–2 together. Perianth segments 5 or 6, green with broad scarious margins, enlarged in fruiting. Stamens 3; filaments entirely connete. Styles bifid at the apex. Fruits globose, trigonous, depressed at the apex. Seeds 3-gonous, with 5–7 sub-parallel longitudinal ribs (Mitra and Jain, 1985).

*P. niruri* Linn. (synonyms: *Diasperus chlorophaeus (Baill)*. Kuntze. *Nymphanthus niruri (L). Lour.*, *P. carolinianus Blanco*, *P. chlorophaeus Baill.*, *P. parvifolius Steud.*, *P. purpurascens Kunth.*, *P. rosellus* (*Müll.Arg*). *Müll.Arg.*, *P. williamsii Standl*). It is a small erect annual herb growing up to 30–40 cm in height and is indigenous to the Amazon rainforest and other tropical areas, including South East Asia, Southern India, and China. Its leaves are 7–12 cm long, and they are alternate, sessile oblong. It has small off-white-greenish flowers, which are solitary, auxiliary, pedicellate, apetalous, and monoecious (Mitra and Jain, 1985). *P. niruri* is closely related to *P. amarus* in appearance and phytochemical contents, but a recent cladistic analysis indicated that the *Phyllanthus* genus is paraphyletic, and therefore, these two problematic and confusing species, *P. niruri* and *P. amarus*, are two individual species (Lee et al., 2006).

*P. urinaria* L. (synonym: *P. leprocarpus* Wight) is widely distributed in pantropic areas. It is a monoecious and short-lived perennial herb and may grow up to 60 cm tall. Erect, annual, glabrous herbs; leaf-bearing branchlets short, flattened, or slightly winged. Leaves distichous, closely placed. Flowers minute, axillary, solitary, subsessile. Stamens 3; filaments connete into a column. Fruits depressed globose, echinulate, scarcely lobed. Seeds 3-gonous, rounded on back, transversly rugose (Kuttan and Harikumar, 2011).

*P. emblica* L. (synonym: *Emblica officinalis* Gaertner). Distributed throughout tropical and subtropical India, wild or planted. It is a deciduous tree. The tree is small to medium in size, reaching 1–8 m in height. Young branch lets reddish brown, hairy, leaves apiculture obtuse, rounded, or chordate at base, glabrous beneath, about 18 × 1.2 cm. Flowers are greenish yellow, usually monoecious. Fruit fleshy more than 1.5 cm in diameter is nearly spherical, light greenish-yellow, quite smooth, and hard on appearance, with six vertical stripes or furrows. The branchlets are not glabrous or finely pubescent, 10–20 cm long, usually deciduous; the leaves are simple, subsessile and closely set along branchlets, light green, resembling pinnate leaves (Kuttan and Harikumar, 2011).

*P. acidus* L. (synonyms: *P. distichous L.*, *Cicca acida* L., *Cicca disticha* L). is native to Malaya Islands and Madagascar and is found throughout Asia and also in the Caribbean region, Central and South America. It is a deciduous trees reaching 2 to 9 m high. Leaves distichous, light green above, glaucous beneath. Flowers densely clustered and elongated, simple or branched racemes from old, defoliated branches, male flowers numerous, female 1–2, stamens 4, much longer than perianth. Ovary 3–4 locular. Fruits fleshy, depressed globose, 6–8 lobed, pale yellow. Fruits borne in loose clusters are pale yellow or white, waxy, crisp, juicy, and very sour with edible small yellow (Kuttan and Harikumar, 2011).

*P. fraternus* G.L. Webster (synonyms: *P. fraternus* subsp. *togoensis* Jean F. Brunel & J.P. Roux *P. lonphali* Wall.) distributed in Tropical Asia especially in Indian subcontinent and Pakistan. It is an erect, annual plant growing up to 45 cm, occasionally to 60 cm (Balakrishnan and Chakrabarty, 2007).

*P. reticulatus* Poir (synonym: *Kirganelia reticulata* Poir). An Asian species of *Phyllanthus* distributed throughout tropical India. It is a large, glabrous or pubescent, straggling shrubs which grows up to 5 m high. Flowers auxiliary, the male flowers in fascicles of 2–6, the females solitary. Perianth segments 5–6, oblong, alternatively with disk glands. Stamens 5, the inner 3 with their filaments connete into column, the outer 2 free, shorter. Ovary 5–10 locular, styles 3, minute. Fruits fleshy, depressed globose, dark purple or black, shining. Seeds 8–10, trigonous granulate (Kawakita and Kato, 2009).

*P. simplex* Retz. (synonyms: *P. virgatus* G. Forst). Distributed in South Asia; India, Sri Lanka to SE. Asia, S. China, Indo-China, and Malesia to Pacific Islands. Fairly common along roadsides, in open fields and dry deciduous forests. Erect or diffuse perennial herb, up to 20 cm high. Leaves distichous, subsessile. Flowers usually solitary, on slender axillary pedicels. Perianth segments oblong, obtuse, slightly enlarged in fruit. Stamens 3, distinct; anthers didymous. Style short, bifid. Fruits globose, 3-lobed. Seeds trigonous, rounded on back, covered with minute tubercles in irregular lines, dark brown (Kuttan and Harikumar, 2011).

## Ethnomedicinal Values

Several *Phyllanthus* species have been utilized for a long time by traditional healers of India, China, Brazil, and countries in the Southeast Asian regions as potent ethnomedicines to treat numerous diseases including those related to the human immune system. *P. amarus*, *P. emblica*, *P. niruri*, *P. reticulatus*, *Phyllanthus corcovadensis*, and *P. fraternus* are among others that are widely used as ethnomedicines in many parts of the world. In Brazil and in many South American countries, the infusion of roots, stems, and leaves of most *Phyllanthus* species have been used to cure a broad spectrum of diseases including intestinal infections, hepatitis B, diabetes, kidney, and urinary bladder disturbances (Calixto et al., 1998). In Asia, several *Phyllanthus* species are used as febrifuge, diuretic, deobstruent, stomachic, and antiseptic. Ayurveda uses the most number of *Phyllanthus* species where 15 species have been used in the management of genitourinary, hypertension, cancer, skin, digestive, hepatic, and respiratory disorders (Adil et al., 2010; Nain et al., 2012; Sarin et al., 2014). Among these, *P. niruri* is the mostly used for the remedy of inflammation, fever, malaria, lithiasis, gonorrhea, and hepatitis. *P. amarus* is used widely in Ayurvedic medicine for the treatment of stomach, liver, kidney, genitourinary system and spleen problems, menorrhagia, gonorrhea, and other genital infections. It is also beneficial in intermittent fevers, diarrhea, ulcers and wounds, gastropathy, dysentery, ophthalmopathy, and scabies (Bagalkotkar et al., 2006; Ifeoma et al., 2013). In Thailand, six *Phyllanthus* species are used extensively as ethnomedicines. *P. urinaria,* *P. amarus*, and *P. virgatus* are used for the treatment of gonorrhea, diabetic, jaundice, and hepatic disorders while the other species, *P. niruri*, *P. acidus*, and *P. reticulatus* are used to treat hypertensive, malarial fever, constipation, and skin and urination disorders. In China, five species are widely used in Tibetan and Traditional Chinese Medicine to treat anuria, dropsy, hypertension, sore throat, and hepatitis B as well as blood and bile disorders. Particularly, *P. niruri*, *P. simplex*, and *P. reticulatus* are used for the treatment of inflammation, urinary infection, rheumatism, and ophthalmic diseases (Nain et al., 2012). In Africa, six *Phyllanthus* species are used to treat tetanus, malarial fever, and wound (Omulokoli et al., 1997; Ifeoma et al., 2013). Specially, in Cameroon, Ghana, Ivory Coast, Kenya, Nigeria, and Zambia, the stem, root, and leaves of various species of *Phyllanthus* are used for the remedy of tetanus, wound, fever, and sexually transmitted diseases.

## Phytochemistry

Many phytochemical, biological, and pharmacological studies of *Phyllanthus* species have been performed due to tremendous ethnomedicinal and therapeutic potentials of the plants. More than 510 compounds have been isolated and identified from several *Phyllanthus *species. Most of the species were reported to comprise of diverse combinations of secondary and bioactive metabolites including lignans, triterpenes, sterols, alkaloids, flavonoids, ellagitannins, and other polyphenols. These compounds provide important medicinal properties to the plants. There are already a few reviews on the phytochemistry and pharmacological properties of *Phyllanthus* species (Calixto et al., 1998; Nahar et al., 2011; Patel et al., 2011; Sarin et al., 2014). Specially, the lignans and tannins isolated from genus *Phyllanthus* have been evaluated for their wide variety of biological activities. Corilagin (**1**), geraniin (**2**), and gallic acid (**3**) are the three most predominant metabolites found in this genus. Mostly, corilagin (**1**), geraniin (**2**), gallic acid (**3**), phyllanthin (**4**), hypophyllanthin (**5**), ellagic acid (**6**), phyltetralin (**7**), niranthin (**8**), catechin (**9**), quercetin (**10**), astragalin (**11**), and chebulagic acid (**12**) are the bioactive metabolites that have been intensively investigated and reported to exhibit diverse biological and pharmacological effects including immunomodulatory activities (Jantan et al., 2014). **Table 1** highlights the similarities and differences in phytochemical composition of the *Phyllanthus* species discussed in this review: *P. amarus*, *P. emblica*, *P. urinaria*, *P. niruri*, *P. acidus*, *P. fraternus*, *P. reticulatus*, and *P. simplex*.

**Table 1:** Phytochemicals isolated from *Phyllanthus* species.

Species	Class	Secondary metabolites	References
*P. amarus,*	Lignans Flavonoids Tannins Alkaloids Triterpenes	Phyllanthin, hypophyllanthin, lintetralin, niranthin, nirtetralin, isolintetralin, isonirtetralin, phyltetralin, hinokinin Rutin, kaempferol, astragalin Gallic acid, ellagic acid, gallotannin, geraniin, corilagin, isocorilagin, amariin, furosin, geraniinic acid, amarinic acid, elaeocarpusin, *Phyllanthusiin A., B., C.*, and *D*. Phyllanthine, securinine, tetrahydrosecurinine, dihydrosecurinine, securinol, allo-secutine, nor-securinine, isobubbialine, epibubbialine, 4-hydroxysecurinine. Lupeol, phyllanthenol, phyllanthenone, phyllantheol, amarosterol A, amarosterol B.	Foo and Wong, 1992; Foo, 1995; Calixto et al., 1998; Kassuya et al., 2006; Singh et al., 2009; Patel et al., 2011; Jantan et al., 2014.
*P. niruri,*	Lignans Flavonoids Tannins Alkaloids Triterpenes	Phyllanthin, hypophyllanthin, niranthin, nirtetralin, phyltetralin, hinokinin, isolintetralin, nirurin, Rutin, astragalin, quercetin, quercitrin, isoquercitrin, kaempferol-4’-rhamnopyranoside, fisetin-4-O-glucoside Geraniin, corilagin, gallic acid, ellagic acid Nirurine, 4-methoxy-nor-securinine, ent- norsecurinine Lupeol, lupeol acetate, phyllanthenol, phyllanthenone, phyllantheol	Mulchandani and Hassarajani, 1984; Joshi et al., 1986; Ueno et al., 1988; Singh et al., 1989; Huang et al., 1992; Calixto et al., 1998.
*P. urinaria,*	Lignans Flavonoids Tannins	Phyllanthin, hypophyllanthin Astragalin, kaempferol, quercetin, quercitrin, isoquercitrin, rutin Geraniin, gallic acid, ellagic acid,	Okuda et al., 1980; Negi and Fakhir, 1988; Satyan et al., 1995; Jantan et al., 2014.
*P. emblica,*	Flavonoids Tannins Diterpenes	Chebulic acid, chibulinic acid, rutin, leucodelphinidin, Ellagic acid, gallic acid, ethyl gallate, corilagin Gibberellin A-1, gibberellin A-3, gibberellin A-4, gibberellin A-7, gibberellin A-9 Zeatin, zeatin nucleotide, zeatin riboside	Srivastava and Ranjan, 1967; Ram and Rao, 1976; Ram and Rao, 1978.
*P. acidus,*	Lignan Triterpenes	Phyllanthol Lupeol, phyllanthol, β-amyrin	Sengupta and Mukhopadhyay, 1966; Pettit et al., 1982.
*P. fraternus,*	Lignans	Phyllanthin, hypophyllanthin, niranthin, nirtetralin, phyltetralin and E,E-2,4-octadienamide,E,Z2,4-decadienamide	Sittie et al., 1998; Sebastian and Setty, 1999; Tripathi et al., 2006; Kavit et al., 2013.
*P. reticulatus*	Tannins Triterpenes	Pyrogallic acid, ellagic acid Friedelin, friedelanol, betulinic acid, glochidonol, 21-α-hydroxy-friedelin	Neves and Neves, 1966; Hui et al., 1976.
P. simplex	Alkaloids Lignans	Phyllanthine, simplexine Hinokin, hypophyllanthin, niranthin, isolintetralin, virgatusin, nirtetralin, phyltetralin	Negi and Fakhir, 1988; Huang et al., 1996.

## Immunomodulatory Effects of *Phyllanthus* Species and Their Bioactive Metabolites

**Figure 3:**
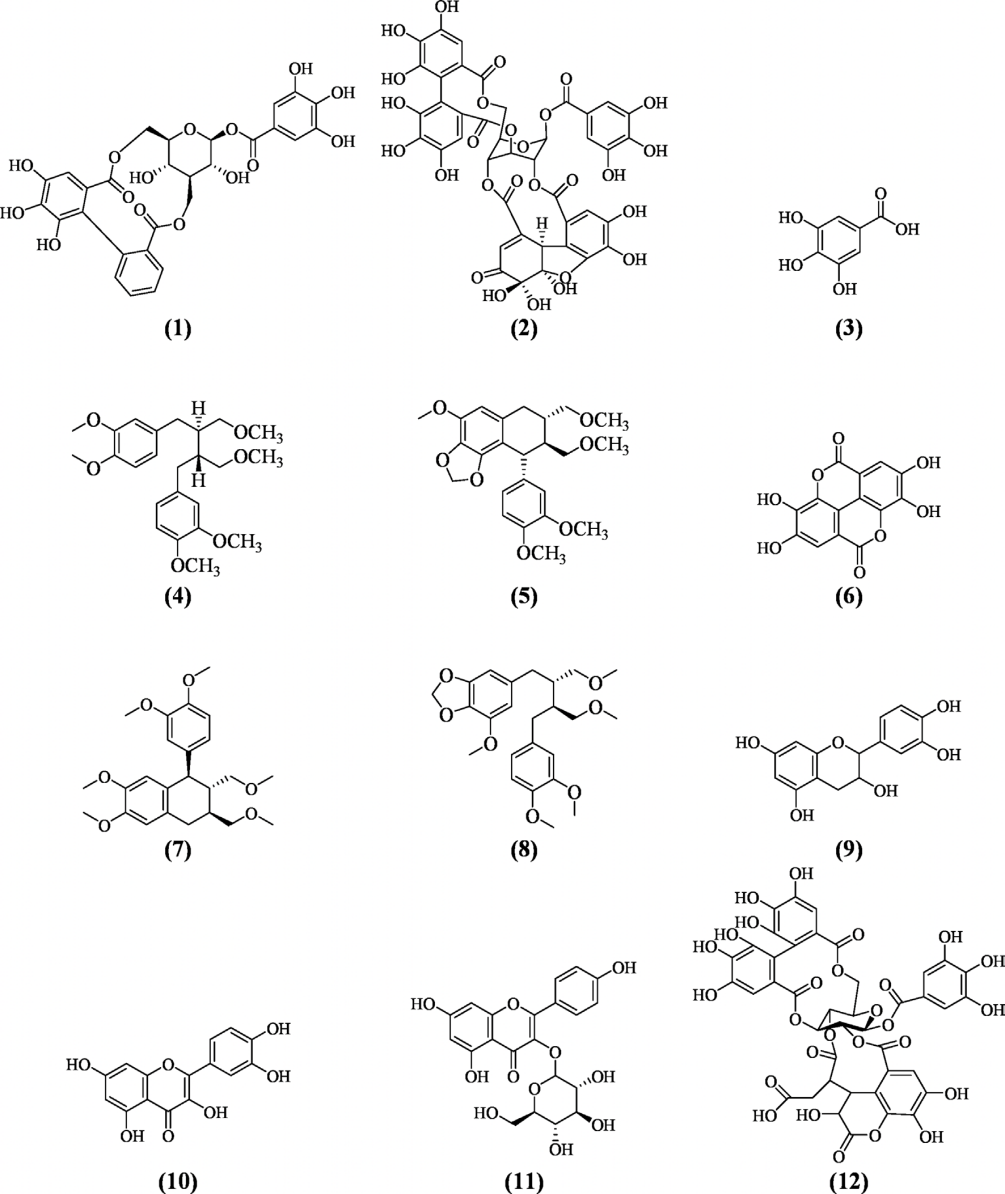
Major bioactive metabolites with potent immunomodulatory properties identified in *Phyllanthus* species.

*Phyllanthus* species have been widely investigated for various biological and pharmacological activities including antioxidant, antiviral, antibacterial, anti-inflammatory, anticancer, hepatoprotective, antimalarial, antiplasmodial, antidiabetic, nephroprotective, hypolipidemic, and diuretic properties (Calixto et al., 1998; Sarin et al., 2014). *Phyllanthus* species have gained much interest among researchers for its immunomodulatory properties through various mechanisms. Many *in vitro* and *in vivo* studies have been performed on various extracts of *Phyllanthus* species and bioactive constituents to evaluate their immunomodulating effects (Ilangkovan et al., 2016a; Harikrishnan et al., 2018a; Harikrishnan et al., 2018b; Harikrishnan et al., 2018). However, most reports on the biological and pharmacological studies of the plants were based on crude extracts which were not standardized. The extracts were not chemically characterized, and the contributions of their chemical constituents to the bioactivities were not clearly correlated and identified. However, some of the extracts were analyzed qualitatively and quantitatively by using validated high performance liquid chromatography (HPLC) methods (Jantan et al., 2014; Ilangkovan et al., 2016b). Some of the compounds especially the lignans and flavonoids and some phenolic compounds have been isolated and identified based on their physicochemical properties and spectroscopy. All the *Phyllanthus* species selected in this review and their bioactive compounds have been determined over the years to possess significant immunomodulating properties. The immunomodulatory properties of the plants are critically discussed in this review. The underlying mechanisms of action of the major bioactive metabolites isolated from different *Phyllanthus* species on the immune system are summarized in **Table 2**. The chemical structures of compounds isolated from these plants with pronounced immunomodulating properties are shown in **Figure 3**.

**Table 2:** The mechanisms of immunomodulatory effects of several bioactive metabolites present in *Phyllanthus* species.

Compound No.	Name of compounds	Species	Mechanism of action	Reference
1	Corilagin	*P. amarus, P. emblica, P. niruri, P. urinaria, P. reticulatus, P. virgatus*	Inhibits MPO, MDA, and translocation of NF-κB and inhibits ROS and NO production Inhibits fMLP-induced chemotaxis in human PMN cells	(Jantan et al., 2014)
2	Geraniin	*P. amarus, P. urinaria, P. emblica, P. wightianus*	Inhibits production of ROS and NO Inhibits pro-inflammatory cytokine release, Inhibits PHA-induced lymphocyte proliferation in human PBMCs	(Jantan et al., 2014)
3	Gallic acid	*P. amarus, P. urinaria, P. emblica*	Inhibits production of ROS and NO Inhibits pro-inflammatory cytokine release Inhibits PHA-induced lymphocyte proliferation in human PBMCs	(Jantan et al., 2014)
4	Phyllanthin	*P. amarus, P. niruri, P. urinaria*	Inhibits cellular and humoral immune response in Balb/C mice Inhibits ROS and NO release, inhibits fMLP-induced neutrophil migration Inhibits pro-inflammatory cytokine release Inhibits MyD88-dependent signaling pathways (NF-κB and MAPK)	(Ilangkovan et al., 2016a; Harikrishnan et al., 2018c)
5	Hypophyllanthin	*P. amarus P. urinaria*	Inhibits production of ROS and NO Inhibits pro-inflammatory cytokine release, Inhibits PHA-induced lympocyte proliferation in human PBMCs Inhibits protein and gene levels of COX-2 and downstream signaling products of PGE2, TNF-α, and IL-1β. Suppresses the inhibitors of IκB, Ikkα/β, NF-κB phosphorylation, and IκB degradation human PBMCs	(Jantan et al., 2014; Harikhrisnan et al., 2018)
6	Ellagic acid	*P. amarus, emblica, P. niruri, P. urinaria*	Inhibits fMLP-induced chemotaxis in human PMN cells Inhibits PMA and zymosan-induced ROS release and LPS-induced NO release in macrophages Reduces phagocytic activity of human PMN cells	(Jantan et al., 2014)
7	Phyltetralin	*P. amarus *	Decreased the specific binding of [3H]-PAF in mouse cerebral cortex membranes. Inhibited the increase of myeloperoxidase activity induced by PAF injection in the mouse paw	(Kassuya et al., 2006)
8	Niranthin	*P. amarus, P. niruri*	Enables the switching from a Th2- to a Th1-type immune response in *Leishmania* BALB/c mice infected by *Leishmania donovani* Inhibits protein and gene levels of COX-2 and downstream signaling products of PGE2, TNF-α, and IL-1β. Suppresses the inhibitors of IκB, Ikkα/β, NF-κB phosphorylation, and IκB degradation and suppresses JNK and ERK	(Chowdhury et al., 2012; Harikrishnan et al., 2018c)
9	Catechin	*P. niruri, P. orbicularis*	Inhibits ROS release Augments expression of ICAM-1 and VCAM-1 Inhibits COX-2 and iNOS release in human chondrocytes	(El-Aziz et al., 2012)
10	Quercetin	*P. emblica, P. urinaria, P. virgatus*	Inhibits *in vitro* LPS-induced TNF-α	(Comalada et al., 2005)
11	Astragalin	*P. urinaria*	Inhibits NO release, IL-6, and PGE-2 by LPS-stimulated RAW 264.7 cells	(Lee et al., 2011)
12	Chebulagic acid	*P. emblica, P. myrtifolius *	Inhibits T-cell proliferation and the killing activity of CD8+ CTL, inhibits VEGFA-induced vascular permeability	(Hamada et al., 1997)

### *Phyllanthus amarus* Schum and Thonn.

#### *In Vitro* Immunosuppressive Effects of *P. amarus*

The standardized methanol extracts of *P. amarus* and *P. urinaria* and their marker compounds, phyllanthin (**4**), and hypophyllanthin (**5**) have been investigated for their inhibitory effects on chemotaxis, phagocytosis, and chemiluminescence of human neutrophils (Yuandani et al., 2013). Among the extracts, *P. amarus* from Malaysia showed the strongest inhibition on chemotaxis of polymorphonuclear leukocytes (PMNs) at a dose of 10 μg/ml, with an IC_50_ value of 1.1 μg/ml, while phyllanthin (**4**) and hypophyllanthin (**5**) showed relatively strong activity with IC_50_ values of 3.2 and 4.4 μg/ml (7.6 and 10.2 μM), respectively. Among all samples tested, phyllanthin at 50 μg/ml showed the strongest inhibition of engulfment activity of phagocytizing cells, at 14.2 and 27.1% for neutrophils and monocytes, respectively. The low percentages of phagocytizing cells signify that the samples were inhibiting the phagocytic cells by reducing the percentage of FITC-labeled *Escherichia coli* ingestion. Phyllanthin (**4**) also showed the lowest IC_50_ value (7.6 μM) among the samples and was a more potent inhibitor of ROS generation than the positive control, aspirin (IC_50_ 10.5 μM). The results indicate that phyllanthin (**4**) might be one of the major contributors to the inhibitory effects of the plant extracts on phagocytic activity of neutrophils. In another study, correlation between the major constituents of the plants (corilagin [**1**], geraniin [**2**], gallic acid [**3**], phyllanthin [**4**], hypophyllanthin [**5**], and ellagic acid [**6**]) with their suppressive effects on phagocytic activity of human neutrophils indicated that geraniin (**2**) at 10 μg/ml was the most active in inhibiting chemotaxis of monocytes and PMNs, with IC_50_ values of 1.69 and 1.09 μM, respectively, both lower than that of ibuprofen, the positive control (Jantan et al., 2014). Corilagin (**1**) and geraniin (**2**) at 12.5 μg/ml demonstrated significant suppression on the reactive oxygen species (ROS) activity with IC_50_ values lower than that of aspirin. The study also showed that the strong inhibition against CD18 expression on leukocytes was mainly due to phyllanthin (**4**) and hypophyllanthin (**5**) which exhibited significant inhibitory effect. Phyllanthin (**4**) and hypohyllanthin (**5**) at 50 μg/ml exhibited strong inhibition with percentage of CD18 expression of 61.56 and 67.17% for monocytes and PMNs, respectively. Yuandani et al., (2016) reported that, among the compounds isolated from *P. amarus*, 1,7,8-trihydroxy-2-naphthaldehyde and ethyl 8-hydroxy-8-methyl-tridecanoate at 50 μg/ml showed strong inhibition on NO production with IC_50_ values of 1.07 and 0.91 μM, respectively. Phyltetralin (**7**) and phyllanthin (**4**) (50 μg/ml) showed strong suppression on lymphocyte proliferation, exhibiting IC_50_ values of 1.07 and 1.82 μM, respectively. Of all the compounds, corilagin (**1**) at 100 μg/ml significantly inhibited TNF-α release with an IC_50_ value of 7.39 μM and, at similar concentration, geraniin (**2**) exhibited the strongest inhibitory activity on interleukin-(IL) 1β release with an IC_50_ value of 16.41 μM.

**Figure 4:**
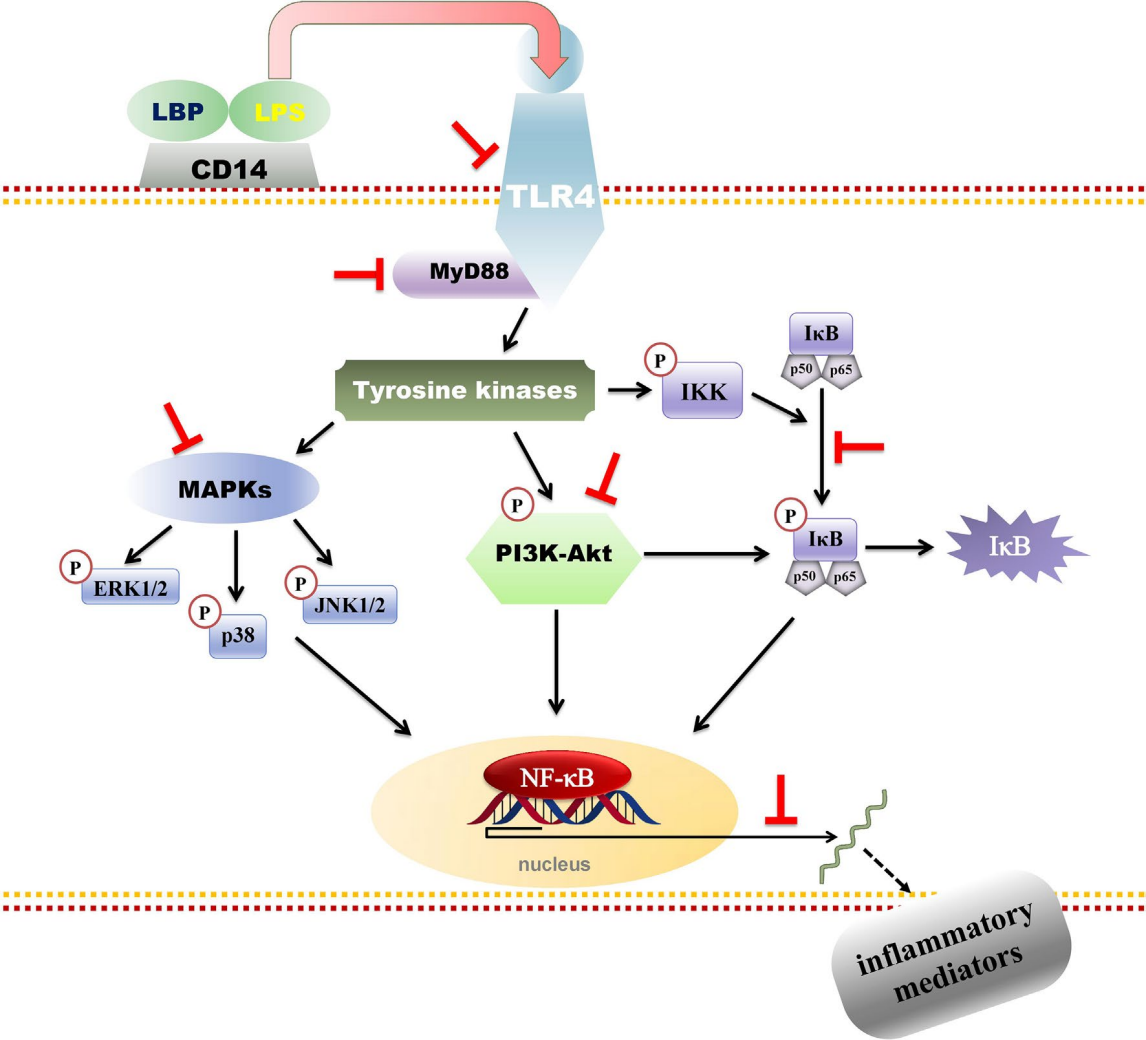
Phyllanthin-mediated inhibition of lipopolysaccharide (LPS)-induced inflammatory responses through NF-κB, MAPKs, and PI3K-Akt signaling pathways in human macrophages.

Recently, Harikrishnan et al., (2018a), Harikrishnan et al., (2018b), and Harikrishnan et al., (2018) investigated the effects of 80% ethanol extract of *P. amarus* and its main constituents, phyllanthin (**4**), hypophyllanthin (**5**), and niranthin (**8**) (24–1.5 μM), using LPS-induced U937 human macrophages. They reported that their anti-inflammatory effects were by downregulating the nuclear factor kappa-B (NF-κB), mitogen-activated protein kinase (MAPK), and phosphatidylinositol-3-kinase (PI3K-Akt) signaling pathways. **Figure 4** depicts phyllanthin-mediated inhibition of LPS-induced inflammatory responses through NF-κB, MAPKs, and PI3K-Akt signaling pathways in human macrophages. The authors evaluated prostaglandin E_2_ (PGE_2_), tumor necrosis factor (TNF)-α, and interleukin (IL)-1β release. In addition, COX-2 levels and MAPK molecules (JNK, ERK, and p38 MAPK) and NF-κB and Akt activations were determined by immunoblot technique while, by using the qRT-PCR, the gene expression levels of COX-2 and pro-inflammatory cytokines were assessed. The results demonstrated that *P. amarus* extract considerably repressed the aforementioned pro-inflammatory mediators’ release and expression of COX-2 protein. Also, the raised mRNA transcription of pro-inflammatory markers was prominently reduced. Furthermore, it downregulated the NF-κB (p65), IκBα, and IKKα/β phosphorylations and reinstated the degradation of IκBα, while increased the expression of Akt, JNK, ERK, and phosphorylation of p38 MAPKs dose-dependently (Harikrishnan et al., 2018a). Additionally, the extract and isolates downregulated the expression of upstream signaling molecules including MyD88 and TLR4 which are essential for the activation of NF-κB, MAPKs, and PI3K-Akt signal transducing pathways. Subsequent studies revealed that phyllanthin (**4**), hypophyllanthin (**5**), and niranthin (**8**) strongly suppressed the protein and gene levels of COX-2 as well as the downstream production of TNF-α, PGE2, and IL-1β. In addition, phyllanthin (**4**) and hypophyllanthin (**5**) attenuated the extracellular signal-regulated kinase (ERK), phosphorylation of c-Jun N-terminal kinase (JNK), and p38 while niranthin (**8**) only inhibited JNK and ERK but did not show any potent effect on p38. The authors concluded that phyllanthin (**4**), hypophyllanthin (**5**), and niranthin (**8**) downregulated TNF-α, COX-2, and IL-1β gene expressions in U937 macrophages by interfering with the activation of MAPKs, NF-κB, and Akt (Harikrishnan et al., 2018a; Harikrishnan et al., 2018b).

The ethanol/water and hexane extracts of *P. amarus* at different dose ranges have been reported to possess anti-inflammatory potential for their inhibitory activity on iNOS, cytokines, and COX-2 *via* the NF-kB pathway (Kiemer et al., 2003). The extracts inhibited LPS-induced production of NO and PGE_2_ in rat Kupffer cells (KC) and RAW 264.7 macrophages. The extracts also attenuated expression of iNOS and COX-2 expression and reduced activation of NF-kB. *P. amarus* inhibited induction of IL-1β, IL-10, and interferon-g in human whole blood. The extracts inhibited the LPS-induced secretion of TNF-α in RAW264.7 and human whole blood, and in galactosamine-sensitized mice. This LPS-induced hepatitis model leads to a large increase in macrophage/KC TNF-α production (Gorgen et al., 1992). In this experimental design, *P. amarus* extract (45 mg/kg) was administered intra-peritoneally (i.p.) to male BalbA2 mice (30 g BW), followed 30 min later by injection of 500 mg/kg galactosamine i.p. and immediately afterwards by 1.5 mg/kg of LPS. Serum sample was obtained by tail bleeding after 90 min. The *in vitro* and *in vivo* data on the inhibition of TNF-α production provide evidence that *P. amarus* extracts exert anti-inflammatory action.

In another study, tumor lysate (TLY) generated from apoptotic stimulation of *P. amarus* extract (1,000 µg/ml) on HCT 116 and MCF-7 cancer cell lines was evaluated for its ability to induce anti-tumor immune response (Mohamed et al., 2018). The TLY-impulsed dentritic cells (DC) to evaluate the latter’s effect on their cellular immune functions including antigen-presenting capacity (APC), phagocytic activity, migration capacity, proliferation of T lymphocytes, release of cytokines, and relative gene expression levels. The results showed that the TLY significantly raised the expression levels of MHC class I, CD 83^+^, 86^+^, and CD 11 c, in pulsed DCs. The levels of IL-12 and IL-6 were increased by TLY-DCs at a ratio of 1:3 (DC; tumor apoptotic bodies); however, it was found that the level of IL-10 was reduced. Also, the phagocytic capacity was highly reduced whereas the chemotaxis ability and proliferative activity of lymphocytes of loaded DCs were significantly increased. In TLY-pulsed dendritic cells, IL-6 and IL-12 showed amplified gene expression of mRNA in comparison to unloaded and LPS-treated DCs. Therefore, the authors suggested that, for a renowned tumor antigen, *P. amarus*-generated TLY has the capacity to prompt an *in vitro* anti-tumor immune response (Mohamed et al., 2018).

The major constituents of essential oil of *P. amarus* were reported to possess immunomodulatory properties. Previous studies have revealed that linalool at a dose range of 10–40 mg/kg has significantly enhanced tumor treatments and provided novel starting points for future anti-cancer research (Li et al., 2014). Whereas, phytol at 50 mg/kg has been reported for its ability to modulate transcription of cells through transcription factors peroxisome proliferator-activated receptors (PPAR-α) and retinoid X receptors (RXR) (Bos et al., 2002). These receptors are subsets of nuclear hormone receptor superfamily and are activated by different lipid species in regulating inflammatory responses. Whereas, geraniol inhibited the inflammation by enhancing the release of IL-10. Besides, 1,8-cineole, a monoterpene oxide found in essential oil of *P. amarus*, possesses anti-inflammatory properties. 1,8-Cineole has substantially reduced the MPO activity in trinitrobenzenesulfonic acid (TNBS)-induced colitis model in rats. In another study, a-terpineol has been reported for its ability to impede inflammatory mediators release in human macrophages stimulated by LPS (Gershenzon and Kreis, 1999).

#### *In Vivo* Immunomosuppressive Effects of *P. amarus*

The effects of treating Wistar-Kyoto rats orally with 80% ethanol extract of *P. amarus* on various cellular immune parameters, including leukocytes migration, Mac-1 expression, phagocytic activity, T and B cell proliferation in Wistar-Kyoto rats have been documented (Ilangkovan et al., 2015). Other cell-mediated activities of *P. amarus* were also studied (CD4^+^ T cells and CD8^+^ T-cells) using mononuclear cells isolated from spleen and serum cytokine production by activated T-cells. *Ex vivo* stimulated neutrophils isolated from rats treated with *P. amarus* (100 to 400 mg/kg) for a period of 14 days have shown substantial dose-dependent suppression of Mac-1 expression, phagocytic activity, and neutrophil migration. Correspondingly, in rats sensitized by sheep red blood cells (sRBC), the treatment of *P. amarus* caused a significant dose-dependent reduction in the proliferation of T and B lymphocytes induced by concanavalin A and LPS, respectively. A noteworthy inhibition of the percentage of CD4^+^ and CD8^+^ expression on spleen cells and in serum cytokines of IL-2 and IFN-γ and IL-4 was seen (Ilangkovan et al., 2015) at a dose of 400 mg/kg of the extract. Interestingly, it was observed that *P. amarus* administration raised the levels of cellular GSH and GST, hence reducing the detrimental effects of cyclophosphamide metabolites, a conventional immunosuppressive drug. In subsequent study, standardized *P. amarus* extract (50–200 mg/kg) effects on cellular and humoral immune responses in Balb/C mice were investigated (Ilangkovan et al., 2016a). The myeloperoxidase (MPO) activity, phagocytosis, NO release, serum immunoglobulins, ceruloplasmin and lysozyme levels, and swelling of footpad in delayed-type hypersensitivity (DTH) were measured. There was a significant dose-dependent inhibition of NO release, MPO activity, *E. coli* phagocytic capacity of peritoneal macrophages, ceruloplasmin, and lysozyme levels as well as the release of serum level immunoglobulins in treated mice. The sRBC-induced swelling rate of mice paw in the DTH was also suppressed.

Hexane extract, the lignan-rich fraction (10–300 mg/kg) or the lignans phyltetralin (**7**), niranthin (**8**), and nirtetralin (0.42–43 mg/kg) isolated from *P. amarus*, administered orally to mice markedly inhibited carrageenan (Cg)-induced paw edema and Cg-induced MPO release. Nirtetralin also inhibited the Cg-induced tissue level of IL-1β (Kassuya et al., 2005). In addition, niranthin (**8**) and nirtetralin (30 nmol/paw) inhibited platelet-activating factor (PAF)-induced paw edema formation in mice (Kassuya et al., 2006). PAF is a naturally occurring phospholipid generated by several types of immune cells including monocytes, macrophages, PMNs, and lymphocytes. PAF has been known to play central role in maintaining several immunological cellular events including increased intracellular calcium level (Schulam et al., 1990), liberation of arachidonic acid (Schulam et al., 1991) phosphoinositide turnover, and up-regulation of the proto-oncogenes c-FOS, c-JUN, and EGR2, as well as the induction of tyrosine phosphorylation of phospholipase C. Several other studies suggested that PAF can regulate cellular events such as cAMP generation (Patke et al., 1994), T cell proliferation (Ilangkovan et al., 2015), and release of immunoglobulins. Thus, it can be suggested that these bioactive constituents of *P. amarus* could potentially downregulate various immune pathways mediated through its direct antagonistic action on the PAF receptor-binding sites.

Phyllanthin (**4**) isolated from *P. amarus* has been evaluated for its immunosuppressive effects on various humoral and cellular immune responses in Balb/C mice, following similar experimental design as above (Ilangkovan et al., 2016b). The compound suppressed cellular immunity *via* inhibition of T and B cell proliferation, differentiation of T-cells, and regulation of cytokines by Th1/Th2 cells and also by inhibition of DTH reaction in sRBC-immunized Balb/C mice. At 100 mg/kg phyllanthin (**4**) exhibited comparable reduction of CD4^+^ and CD8^+^ percentage expression in spleen cells to that of cyclosporin A at 50 mg/kg. In DTH reaction, phyllanthin (**4**) at 100 mg/kg also exhibited profound suppression of swelling rate of mice paw induced by sheep red blood cells. The serum levels of ceruloplasmin and lysozyme were significantly inhibited in mice fed with 40 and 100 mg/kg of phyllanthin. Phyllanthin (**4**) also downregulated anti-sRBC immunoglobulins (IgM and IgG) antibody titer in immunized and phyllanthin-treated mice in a dose-dependent manner with maximum inhibition at 100 mg/kg. An increase in the antibody levels of serum may be attributed to the increased receptiveness of macrophages, as the antibody production was strongly associated with the activated macrophages, T cells, and B cells. Macrophages serve as antigen-presenting cells (APCs) and have the ability to phagocytose and digest the foreign antigen into fragments as well as present to T-lymphocyte and B-lymphocytes with the aid of surface major histocompatibility class II (MHCII) molecules (Janeway et al., 2005).

Another study reported preventive anticancer activity of *P. amarus* extract against cyclophosphamide (25 mg/kg BW, i.p.)-induced toxicity in mice. Oral administration of 75% methanol extract of *P. amarus* (250–750 mg/kg BW) resulted in significant reduction of myelosuppression and enhanced bone marrow cellularity, and number of white blood cell (WBC) count including monocytes in mice (Kumar and Kuttan, 2005). The study showed that oral administration at a high dose of 750 mg/kg of *P. extract* alone did not cause adverse effect to the mice. Several immune system studies (*in vitro and in vivo*) revealed that extensive research has been performed to explore the immunomodulatory effects of *P. amarus* using cells of the immune system: neutrophils, macrophages, and lymphocytes (T and B cells). However, still more research is needed to be carried out by exploring the immune-related effects with other immune cells like dendritic cells, natural killer cells, eosinophils, and basophils. Effects of the plant samples on disease models of immune-related and chronic inflammatory disorders should be explored to support the existing reports. No clinical study has been carried out on *P. amarus*.

### *Phyllanthus niruri* Linn.

#### *In Vitro* Immunomodulating Effects of *P. niruri*

A pre-clinical study by Ma’at et al., (1996) demonstrated the ability of the extracts of *P. niruri* (50–200 mg/kg) to attenuate both cellular and humoral immune responses. In addition, Nworu et al., (2010) have shown that *P. niruri* aqueous extract (12.5–200 µg/ml) exhibited pronounced stimulation and mitogenic activities on lymphocytes and macrophages of experimental animals. It has been reported that the extracts of the plant have potentially attenuated pro-inflammatory cytokines (TNF-α) release by LPS-activated macrophages. The extracts of *P. niruri* have also demonstrated a significant enhancement in the expression of CD69 and proliferation of lymphocytes (T and B cells) and also in the release of Th-1 (IFN-γ) and Th-2 (IL-4). This study suggested that the plant extract has the ability to enhance proliferation of T- and B-lymphocytes which could be due to activation of MAPKs. The study has also reported that plant extract inhibited NO release by bone marrow macrophages from treated mice.

*P. niruri* was reported to contain various bioactive phytochemicals, including lignans, flavonoids, terpenoids, alkaloids, polyphenols, coumarins, saponins, and tannins (Bagalkotkar et al., 2006). Among the compounds abundantly identified in the plant, catechin (**9**), quercetin (**10**), and astragalin (**11**) have been reported for their ability to regulate the immune system through various mechanisms. It was described that catechin (**9**) suppressed NF-κB activation stimulated by PMA in Jurkat cells (El-Aziz et al., 2012). Catechin (**9**) has also been reported to inhibit phytohemagglutinin (PHA) or phorbol ester (PMA)-induced lymphocyte proliferation. Additionally, lupeol, a pentacyclic triterpene present in the plant, has been reported to inhibit superoxide release by preventing tyrosyl phosphorylation of a 45kDa protein in human PMNs. Quercetin (**10**) potentially suppressed LPS-induced expression of TNF-α, IL-1β, and IL-6 (pro-inflammatory cytokines) by inhibiting p38 MAPK and ERK activations (Cho et al., 2003). ERK and p38 MAP kinases are protein kinases with different immunological activities (Roux and Blenis, 2004). The activation of these protein kinases plays a major role in COX-2 gene expression upon activation. Hyperexpression of COX-2 enzyme leads to impaired function of immune cells such as T- and B-lymphocytes, dendritic cells in immune-related disorders such as systemic lupus erythematosus (SLE), thyroiditis, rheumatoid arthritis, polymyositis, scleroderma, pulmonary fibrosis, Addison disease, juvenile (type 1) diabetes, and glomerulonephritis (Brudvik and Taskén, 2012). In this context, the ability of the plant and its constituents to downregulate COX-2 expression would be of worthful in the management of autoimmune diseases. Another study revealed the ability of quercetin (**10**) to suppress the raised levels of TNF-α and NO in murine macrophage RAW 264.7 cells activated by LPS. Quercetin (**10**) has also been reported to regulate the secretion of IFN-γ and IL-2 in T-helper cells (Nair et al., 2006). It was reported that quercetin (**10**) could alleviate the effects of viral inflammation based on inhibition of cytokine, NO, chemokines, and growth factors in dsRNA-induced macrophages *via* the calcium-STAT pathway (Kim and Park, 2016). Astragalin (**11**) is a flavanone and has been reported for its ability to modify the immune responses by enhancing phagocytosis capacity of phagocytes by increasing the number of macrophages and promoting the release of antibodies (Desai et al., 2014). It has been shown that astragalin (**11**) inhibited TNF-α, IL-1, and IL-6 through inactivation of NF-κB.

#### *In Vivo* Immunomosuppressive Effects of *P. niruri*

The methanol extract of *P. niruri* was reported to modulate both innate and adaptive immune responses. It has been revealed that the extract of *P. niruri* could potentially regulate cellular immune responses by inhibiting sRBC-induced DTH in mice. DTH is an important cell-mediated immune response and occupies the central role to protect the body against several intracellular pathogens (Bonilla and Oettgen, 2010). This cellular response is primarily mediated by T helper type 1 cells which releases cytokine IFN-γ. This cytokine causes the activation of macrophages and accounts for the symptoms of inflammation. Also, cytotoxic T cells and memory T cells are considered to be involved in DTH reaction (Chen et al., 2009). In addition, administration of the *P. niruri* extract to mice has successfully regulated serum levels of primary and secondary antibodies (Eze, 2014). Another study conducted by Porto et al. (2013) revealed that spray-dried standardized extracts of *P. niruri* have potentially inhibited carrageen-induced edema and thioglycolate-induced neutrophil migration in *Mus musculus* Swiss male mice. Another study evaluated the effects of the plant extract on leukocyte profile of broiler chickens infected with *Mycoplasma gallisepticum*. The experimental analysis showed that the extract could decrease the total number of leukocytes in this animal model (Hidanah et al., 2018). The metabolites profiles of *P. niruri* as any other *Phyllanthus* species change with its geographical distribution due to the changes in soil composition, difference in genetic variation of the plant population growing at altered heights, and other environmental factors. Thus, good quality assurance and appropriate standardization of herbal products play a central part in order to confirm the varying amounts of phytochemical constituents in the plant. Besides, the immunomodulatory effects of *P. niruri* may vary considerably, depending on the experimental conditions used, including the type of extract, animal model, treatment scheme, and different pathological conditions of the experimental model. Dosage and concentration of raw extracts of the plant are crucial in order to achieve the desired benefit.

#### Clinical Studies of *P. niruri* on the Immune System

Although there were insufficient pre-clinical data, two non-systematic clinical investigations of *P. niruri* on pulmonary tuberculosis have been performed. One study demonstrated that the extract (50 mg thrice a day) has significantly elevated plasma IFN-γ and insignificantly downregulated TNF-α level, after continuous administration of *P. niruri* for 2 months. The study has also reported marked elevation in plasma level of TNF-α at the 6th month of treatment. The study claimed that the ability of the plant extracts to increase the IFN-γ, IL-6, and TNF-α production by leukocytes enabled eradication of mycobacteria in tuberculosis (TB) by inducing the release of NO. In addition, the extracts of *P. niruri* have also been reported to regulate Th 2 lymphocytes (Radityawan, 2005). In another study, the plant extract in the presence of standard TB regimes has been reported to produce adequate inhibition in the release of IL-10 in a double-blind, randomized, placebo-controlled study conducted in patients identified with TB with moderate to severe lesion (Halim and Saleh, 2005). Putri et al. (2018) used an *in vitro* approach to explore *P. niruri* potential in stimulating the immune cells activity in TB patient. Therefore, immune system cells like peripheral blood mononuclear cells and macrophages were collected from pulmonary TB patients and treated with different doses of *P. niruri* aqueous extract, and the parameters i.e., NO release, phagocytosis, and proliferation of cells were assessed. The results showed that plant extract prompted the proliferation of PBMCs, raised NO levels release and enhanced phagocytosis by macrophages.

### *Phyllanthus urinaria* L.

#### *In Vitro* Immunosuppressive Effects of *P. urinaria*

The effects of *P. urinaria* and its bioactive compounds on the immune system have been described in various studies. The methanol and 80% ethanol extracts of *P. urinaria* and its bioactive compounds (10–0.625 µg/ml) (corilagin [**1**], geraniin [**2**], gallic acid [**3**], phyllanthin [**4**] and hypophyllanthin [**5**], ellagic acid [**6**]) exhibited potent inhibitory activity on the fMLP-induced migration of neutrophils and monocytes. *P. urinaria* (12.5–0.78 µg/ml) has also exhibited inhibition of zymosan- and PMA-stimulated ROS release by neutrophils and monocytes. The ethanol extract of *P. urinaria* prominently reduced the release of IFN-γ, TNF-α, IL-1, IL-2, and IL-6. Besides, the extracts of *P. urinaria* also exhibited inhibitory activity of mitogen-induced B and T cell proliferation (Yuandani et al., 2013). Ilangkovan et al. (2015) have reported that corilagin (**1**) was able to inhibit phagocytic activities of human phagocytes *via* various pathways. The study has reported that corilagin (1) suppressed zymosan- and PMA-induced release of ROS dose-dependently and inhibited fMLP-induced phagocyte migration as well as *E. coli* engulfment by human phagocytes. In addition, it has also been reported for its inhibitory activity on PHA-induced proliferation of T cells and release of LPS-induced pro-inflammatory cytokine release. Corilagin (**1**) substantially inhibited pro-inflammatory cytokines and mediators production including TNF-α, IL-1β, IL-6, NO (iNOS), and COX-2 on protein level as well as gene levels *via* NF-κB pathway inhibition; suppressed the IL-10 release; and promote release of anti-inflammatory factor HO-1 (Zhao et al., 2008). Corilagin (**1**) inhibited NF-kappaB/DNA interactions *via* binding to NF-kB and affected IL-8 gene expression in TNF-alpha-treated IB3-1 cells (Gambari et al., 2012). In another study, it has been shown that corilagin (**1**), gallic acid (**3**), and phyllanthin (**4**) treated RAW 264.7 cells upon stimulation by LPS, suppressed COX-2 expression at both gene and protein levels. In addition, the compound also suppressed NF-κB, an important mediator and served an essential part in the regulation of pro-inflammatory mediator’s expression. Corilagin (**1**) isolated from *P. urinaria* concentration dependently scavenged DPPH and hydroxyl and superoxide radicals. In addition, phyllanthin and gallic acid were also able to significantly repress NO production in isolated rat peritoneum macrophages (Yuandani et al., 2013; Jantan et al., 2014).

#### *In Vivo* Immunosuppressive Effects of *P. urinaria*

The immunomodulatory effects of corilagin (**1**) isolated from *P. urinaria* were also assessed in an *in vivo* study. Corilagin (7.5–30 mg/kg; intraperitoneally) was administered to animals for 7 days as well as challenged with 2% DSS drinking water for 5 days (Xiao et al., 2013). The results revealed that corilagin significantly shortened the colon length, decreased the damage of colon tissue, and suppressed myeloperoxidase activity. Furthermore, the colon tissues of corilagin-treated mice showed the reduced secretion of TNF-α, IL-6, and IL-1β and down-regulated expression of cleaved caspase-3 and cleaved caspase-9. However, to further explore the effects of *P. urinaria* and to justify the existing reports, several studies to develop disease models of certain immune-related and chronic inflammatory disorders have to be carried out.

### *Phyllanthus emblica* L.

#### *In Vitro* Immunosuppressive Effects of *P. emblica*

*P. emblica* is another beneficial *Phyllanthus* species that has been shown to exhibit various biological activities including immunomodulating properties. Tetrahydrofuran, methanol, and 1,4-dioxane extracts of the herb have suppressed LTB4-induced chemotaxis of human PMNs and fMLP-induced release of granules (Moilanen, 1997). The suppressive effects on chemotaxis and human neutrophil’s release of granules were reported to be linked with increased quantity of polar compounds in the extracts. Diethyl ether extract of *P. emblica* potentially suppressed calcium ionophore A23187-induced LTB4 release from human neutrophils and TXB2 release in platelets. Phytochemical analysis of *P. emblica* showed the presence of a high proportion of polyphenols and hydrolysable tannin-derived compounds that naturally possess antioxidant properties (Scartezzini and Speroni, 2000). The bioactive tannins and polyphenols responsible for immunomodulatory properties of *P. emblica* included gallic acid, ellagic acid, corilagin, chebulagic acid (**12**), and quercetin. These and other tannins from *P. emblica* have been demonstrated to regulate the immune system *via* various mechanisms. Chebulagic acid (**12**), a bioactive tannin (10–1,000 µM) found in *P. emblica*, has been reported to inhibit the NF-κB signaling pathway. As stated earlier, NF-κB signaling pathway plays crucial role in modulating the expression of genes influencing a wide array of biological processes including innate and adaptive immunity, inflammation, as well as the development of B-lymphocytes. Impaired regulation of NF-κB may lead to various immune/non-immune-related disorders including cancer, inflammatory, and autoimmune diseases (Hamada et al., 1997). Thus, the immunomodulatory effects of the plant could be its potential to regulate NF-κB signaling pathway. Besides, luteolin and apigenin (5–10 µg/ml) are important flavones found in *P. emblica* and were able to modulate the immune system by attenuating LPS-induced splenocyte proliferation and enhanced humoral immune responses (Wang et al., 2014). The investigation has also shown the ability of the compounds to enhance natural killer (NK) cells and cytotoxic T lymphocytes (CTL) activities. Thus, it can be suggested that the immunomodulatory properties of the extract of *P. emblica* could be due to synergistic effects of these compounds.

In one study, the ethanol extract of *P. emblica* was assessed for its proliferative activities on human cancer cell lines (HeLa, HT29, HCT116, MCF7) using cell cycle arrest, apoptosis induction (flow cytometer) and MTT assay. The results suggested that *P. emblica* fruit extract (0.2–1 mg/ml) was able to avert cancer cell growth through apoptosis and cell cycle arrest. Also, MTT assay showed that the ethanol extract of *P. emblica* dose-dependently reduced cancer cells sustainability as well as the Jurkat cells were the most sensitive to the extract (IC_50_ values ranged 0.12–0.69 mg/ml). Therefore, *P. emblica* fruit as an alternative therapeutic agent for cancer prevention and treatment (Kumnerdkhonkaen et al., 2018) was suggested. PEPW80-1, a novel water-soluble polysaccharide was isolated from the pulp tissues of *P. emblica* and purified by Sephadex G-100 column and Sephacryl S-300 HR chromatography. It was investigated for its immunomodulating and free-radical inhibitory activities *in vitro*. The antioxidant assays showed that PEPW80-1 (0.1–3.2 mg/ml) possesses DPPH and hydroxyl radical-scavenging property, while the immunomodulatory assays showed that PEPW80-1 could enhance the proliferation of mouse splenocytes (Zeng et al., 2017).

#### *In Vivo* Immunosuppressive Effects of *P. emblica*

Administration of the fruit extract of *P. emblica* to alcohol-treated rats suppressed the NO, protein carbonyls, and lipid peroxide levels and enhanced the properties of the antioxidant enzymes including nicotinamide adenine dinucleotide (NADH) dehydrogenase, **succinate dehydrogenase** (SDH), and cytochrome c oxidase (Kumar et al., 2014a). In another study, *P. emblica* was evaluated for the probable anti-inflammatory mechanism accountable for averting tumorigenesis of pre-cancerous lung lesions. Male A/J mouse model was employed for *in vivo* investigation while the parameters investigated include the levels of MIP-2, TNF-α, IL-6, IL-1β, COX-2, hypoxia-inducible factor-1 (HIF-α), miR-101, and Lin28B protein levels. The findings from the study revealed that *P. emblica* extract treatment significantly reduced the raised levels of pro-inflammatory cytokines in lung tissue. In addition, the protein expressions of COX-2 and HIF-α were remarkably downregulated by *P. emblica* extract (5–10 g/kg) treatment. Also, the extract markedly upregulated the expression of miR-101 and downregulated IL-1β and Lin28B levels (Wang et al., 2017).

#### *Phyllanthus acidus* L.

It was reported that *P. acidus* could potentially regulate the immune system by inhibition of superoxide anion scavenging activity (Chakraborty et al., 2012). Superoxide anions are considered the strongest ROS among all other free radicals, and it plays a crucial role in ROS generation such as H_2_O_2_ and singlet oxygen which are associated with cellular damage. The *P. acidus* extracts have also been reported to downregulate NO release. NO is basically released in huge quantity from the amino acid l-arginine by the inducible nitric oxide synthase (iNOS) which is expressed by many immune cells especially macrophages upon exposure to various stimuli including bacterial LPS, cytokine, or viruses (Chakraborty et al., 2012). The activated macrophages will then release large amount of NO and PGE2 *via* iNOS and COX-2 as well as several pro-inflammatory cytokines including TNF-α, IL-1β, and IL-12 (Haque et al., 2018a; Haque et al., 2018b). These cytokines have great influence in both innate and adaptive immunities. In response to these cytokines, IFN-γ is released by TH1 cells. The release of IFN-γ will subsequently activate macrophages to release NO following enhanced iNOS expression (Berger, 2000). The prevention of the exaggerated release of NO in immune cells through control of regulatory pathways may support the treatment of diseases that are associated with excessive release of NO. Moreover, *P. acidus* extract (50–300 µg/ml) suppressed the expression of iNOS and COX-2 and inhibited the NF-κB. Upstream signaling events of NF-κB translocation, phosphorylation of Src and Syk, and formation of Src/Syk signaling complexes were also downregulated by the methanol extract of *P. acidus* (Hossen et al., 2015).

Several bioactive compounds identified in *P. acidus* were considered to be the fundamental contributors of immunomodulatory properties of the herb including adenosine which is a purine nucleoside. It has been reported that adenosine potentially modulated mononuclear phagocyte functions through 4 G-protein-coupled cell membrane receptors which are known as A1, A2A, A2B, and A3 receptors (Haskó and Pacher, 2012). The binding of adenosine to different types of adenosine and A3 receptors expressed on different innate immune cells enable the compound to modulate innate immune response. Kaemferol (25–100 µM) is yet another bioactive compound found in the extract which has been reported to inhibit the expression of iNOS mRNA in a dose-dependent manner (Rho et al., 2011). It is well known that iNOS assists in the release of NO, and the transcription factor NF-κB inhibits NO release indirectly by affecting the expression of iNOS (Salvemini et al., 1996). Furthermore, a well-known antioxidant has been identified in the extract of *P. acidus*. It has been reported that 4-hydroxybenzoic acid has successfully inhibited cellular immune response by suppressing carrageenan-induced edema in rodents. The compound has also potentially inhibited NO production. Besides, hexadecanoic acid was reported as one of the major volatile constituents from the ripe fruit of *P. acidus*. Hexadecanoic acid has been reported as an inhibitor of phospholipase A2. Phospholipase A2 is responsible for various types of inflammatory and immune-related diseases including asthma, rheumatoid arthritis, atherosclerosis, Crohn’s disease, myocardial infarction, and cancer (Aparna et al., 2012). Although a few potent activities have been reported, the plant lacks proper phytochemical screenings and in-depth mechanistic investigations. Extensive investigation on relevant signaling events followed by *in vivo* studies would be more interesting to elicit the plant potent immunomodulatory properties.

#### *Phyllanthus reticulatus* Poir

The hydroalcohol extracts of *P. reticulatus* fruits and leaves were assessed for immunostimulatory properties on Albino mice at oral doses of 100 and 200 mg/kg. The assessment of immunostimulatory properties on specific immunity and non-specific immunity was investigated by neutrophil adhesion test, carbon clearance test, and cyclophosphamide-induced myelosuppression. The enhancement in the carbon clearance index of the fruit and leaf extracts of *P. reticulatus* revealed the augmentation of the phagocytic activity of mononuclear macrophage (Kumar et al., 2014b). In addition, hydroalcohol extract of *P. reticulatus* has been described to significantly suppress the carrageenan-induced rat paw edema. In an acetic acid-induced writhing test, the ethyl acetate extract of the plant exhibited 51.23 and 65.12% inhibition of writhing at the concentrations of 150 and 300 mg/kg, respectively. A substantial elongation of tail-flick time had been apparent both in the ethyl acetate and methanol extracts (42.38% and 60.49%, respectively) only at the concentration of 300 mg/kg. Moreover, in the carrageenan-induced rat paw edema model, the methanol extract of the plant at the concentration of 300 mg/kg revealed 40.0% inhibition of paw edema after 4 h (Saha et al., 2007). The analgesic and anti-inflammatory effects of the 80% methanol extract of the plant was again demonstrated by a recent investigation lead by Akhter et al. (2018). Acetic acid-induced writhing as well as formalin-induced were performed to assess the analgesic effect of plant extract (100- and 200-mg/kg b.w.) in the tested models (mice) in comparison to the control. The % inhibition of the standard drug and the extract (100 and 200 mg/kg) was at 40.19% and 51.95%, respectively. The anti-inflammatory effects of the extract using the carrageenan-induced anti-inflammatory in mice were performed at doses of 100- and 200-mg/kg b.w. in the tested models compared with the control. Anti-inflammatory activity of the crude extract of plant (100- and 200-mg/kg b.w.) was found in the tested models in comparison to the control (Akhter et al., 2018). These outcomes proved that *P. reticulatus* extract possessed remarkable analgesic and anti-inflammatory activities. The immunomodulatory effects of the bioactive metabolites of the plant should be performed on pro-inflammatory mediators’ release and expression followed by considering several signaling pathways like MAPKs, NF-κB, and other events to provide information on their immunomodulating property at molecular level.

#### *Phyllanthus fraternus* G.L. Webster

*P. fraternus* is quite prevalent in the wet rainforest and closely related to other *Phyllanthus* species including *P. amarus*, *P. niruri*, and *Phyllanthus sellowianus*. The phytochemical analysis *of P. fraternus* plant extract showed the presence of alkaloids, flavonoids, tannins, saponin, glycosides, sterols, and phenols which are the main contributors of pharmacological properties of the plant extract (Sittie et al., 1998; Habib-Ur-Rahman et al., 2004). Phyllanthin, hypophyllanthin, niranthin, nirtetralin, phyltetralin, and E,E-2,4-octadienamide,E,Z2,4-decadienamide are the major metabolites isolated from the herb (Sittie et al., 1998; Sebastian and Setty, 1999; Tripathi et al., 2006). Previous study reported by Sailaja and Setty (2016) has shown the ability of *P. fraternus* extract (100 mg/kg) to inhibit generation of superoxide radicals in allyl alcohol-primed oxidative stress in liver mitochondria (Sailaja and Setty, 2006). Besides, the extracts of *P. fraternus* (100–200 mg/kg) have also been stated to suppress carrageenan-induced paw edema (Oseni et al., 2013). Paw edema induced by carrageenan is a suitable model to investigate the role of inflammatory mediators in acute inflammation (Salvemini et al., 1996). The activation of immune cells such as neutrophils and macrophages contributes to inflammatory responses by releasing oxygen-derived free radicals and NO (Jones et al., 2007). Therefore, the suppressive effect of *P. fraternus* on carrageenan-induced inflammation may be due to its ability to modulate various biological mediators including PGE_2_, histamine, arachidonic acid, NO, and the release of ROS. So far, the investigations were confined to the study on plant extracts. The bioactive metabolites should be taken into consideration in ascertaining the plant bioactivities.

#### *Phyllanthus simplex* Retz.

Chouhan and Singh (2011) reported that the petroleum ether and ethanol extracts of *P. simplex* (100–500 µg/ml) have remarkable scavenging activity of DPPH, hydroxyl, and superoxide radicals. The ethanol extract of the plant potentially suppressed the release of NO from macrophages isolated from rat peritoneum. Additionally, it has also been found to exhibit significant inhibition of carrageenan-induced paw edema as well as inhibition of granuloma formation induced by cotton pellet (Chouhan and Singh, 2011). The phytochemical evaluation of ethanol extract of the plant revealed that presence of phenolics could be the reason of anti-oxidant and anti-inflammatory properties of the plant. Thus, further research should be focused on the isolation and characterization of the phytochemicals especially on phenolics. The research lacks extensive immunological screenings and mechanistic investigation which could be key focus points to conclude the plants potency and effects on the immune-related disorders.

## Conclusion and Future Prospects

The extensive experimental data generated from immunomodulatory studies of *Phyllanthus* species revealed the development of great enthusiasm toward discovering novel constituents from these natural resources that can potentially modulate the immune responses of human system. However, the challenges encountered by the possible application of extracts of various species of *Phyllanthus* and their bioactive compounds as immunomodulators need to be addressed. The inconsistency of pharmacological responses by the plant extracts is a main concern. This can be elucidated through the typically strong reliance of the herb secondary metabolites on environmental signals that can interrupt reproducibility of results with extracts. Besides, the consistency of dosage is also one of the major challenges encountered by researchers as the quantity of bioactive compounds produced may vary due to different geographical regions and environmental factors. These problems can be rectified if the principles of standardization of extracts and enriched fractions are thoroughly imposed in all the investigations. The bioactive compounds of *Phyllanthus* species especially phyllanthin, hypophyllanthin, gallic acid, niranthin, ellagic acid, geraniin, and corilagin are greatly varied in terms of the quantity obtained in different altitudes; therefore, it is challenging to yield analogous quality in every batch. Network pharmacology in combination with “omics” techniques such as genomics, proteomics and transcriptomics, metabolomics, and metabonomics have recently become possible to examine simultaneous molecular effects of chemical compounds present in plant extracts.

Future research on *Phyllanthus* species should focus on identification of bioactive constituents responsible for the immunomodulatory properties of the plant extracts and qualitative and quantitative analyses of chemical markers for standardization and determining the underlying mechanisms of action. Furthermore, paradoxical effects of *Phyllanthus* extracts on immunomodulatory activity have been observed in previous studies. Here, we can postulate that the pathway of action of the extracts may be dependent upon the normal functioning of different organs in a system which works together to absorb, distribute, metabolize, and excrete it at a certain rate. Thus, the contribution of the course of extracts or natural compounds may be different in different pathological condition as normal functioning of organs is disturbed in pathological condition. Though some signaling and molecular level studies have been conducted on *P. amarus*, still, extensive molecular work is needed to be carried out with other species of genus *Phyllanthus*. There is a huge research gap that is required to be filled in order to cover all imperative signaling events concomitant with immune responses that include TLR4, MyD88, ATF2, proteomic and genomic level research on MAPKs and NF-κB pathways as well as kinase activity of Syk, Src, and IRAK1 and promoter activity of AP1, NF-κB, and CREB to justify genus *Phyllanthus* as a persuasive immunomodulatory lead. Although suppressive effects of the plants are promising for their further development into immunosuppressive agents, their potential adverse effects in clinical applications have to be considered. The inhibitory effect of a central transcription factor like NF-kB and MAPK might not only attenuate pro-inflammatory factors but might also reduce induction of other critical factors in the immune response. Thus, the effects of the plants on various other cytokines and mediators should also be investigated. Adequate preclinical data have to be generated before the plant samples can be subjected to clinical trials. Although two clinical studies have been carried out on *P. niruri*, these are preliminary studies with small sample size and sufficient preclinical data have not been generated. Sufficient data generated from preclinical testings which include pharmacokinetic and toxicological studies data have to be available before systematic clinical trials with double-blind, randomized, placebo-controlled studies can be pursued on these plants and their bioactive metabolites.

## Author Contributions

IJ participated in the concept, revised the manuscript and approved the final version to be submitted for publication. AH, MI and LA drafted the manuscript. All authors read and approved the final manuscript.

## Funding

The work was funded by Taylor’s University, Malaysia under the Strategic Research Initiatives (no. SRI-109491).

## Conflict of Interest Statement

The authors declare that the research was conducted in the absence of any commercial or financial relationships that could be construed as a potential conflict of interest.

## Abbreviations

APCs, antigen-presenting cells; BW, bodyweight; CD, cluster of differentiation; Cg, carrageenan; COX, cyclooxygenase; Con-A, concanavalin-A; CTL, cytotoxic T-lymphocyte; DTH, delayed type hypersensitivity; DC, dendritic cells; DPPH, diphenyl-1-picrylhydrazyl; ERK, extracellular signal-regulated kinase; FMPL, formyl methionine phenyl alanine; HIF, hypoxia induced factor; HYP, hypophyllanthin; IFN-γ, interferon-gamma; IL, interleukin; IκBα, inhibitor of kappa B alpha; IKK, IκB kinase; iNOS, inducible nitric oxide synthase; LPS, lipopolysaccharide; MAC-1 macrophage-activated complex-1; MAPK, mitogen-activated protein kinase; MHC, major histocompatibility complex; MIP, macrophage inflammatory protein; MCP-1, Monocyte chemotactic protein-1; MPO, myeloperoxidase; NADPH, nicotinamide adenine dinucleotide; NF-κB, nuclear factor-kappaB; NK, natural killer; NIR, niranthin; NO, nitric oxide; PKC, protein kinase C; PBMCs, peripheral blood mononuclear cells; PAF, platelet-activating factor; PGE_2_, prostaglandin E_2_; PPAR-alpha, peroxisome proliferator-activated receptor-alpha; PHA, phytohaemagglutinin; PMA, phorbol 12-myristate 13-acetate; ROS, reactive oxygen species; PMNs, polymorphonuclear neutrophils; RXR, retinoid X receptor; SDH, succinate dehydrogenase; SLE, systemic lupus erythematosus; sRBCs, sheep red blood cells; TL, tumor lysate; TLR, toll-like receptor; TNBS, trinitro benzene sulfonic acid; TNF, Th-cells, T helper cells; tumor necrosis factor; WBC, white blood cell.
